# JUCars-2024: A dataset of used car listings in Jordan

**DOI:** 10.1016/j.dib.2026.112682

**Published:** 2026-03-16

**Authors:** Nisrean Thalji, Khalid Tahat

**Affiliations:** a Department of Smart Systems, Al-Balqa Applied University, Jordan; b Faculty of Computer Studies, Arab Open University, Amman, Jordan

**Keywords:** Used car market, Vehicle pricing, Automotive dataset, Web scraping, Machine learning applications, Jordanian market dynamics, Vehicle attributes

## Abstract

The used car market in Jordan is a very important component of national transportation and economic systems that reflect consumer preferences, import policies, and affordability constraints. This article presents the Jordanian Used Cars Dataset (JUCars-2024), records of used car advertisements collected throughout 2024. The dataset contains detailed information about vehicles offered for sale, their prices, technical features, and location-related features. Online car marketplaces in Jordan were selected as prominent online car markets and used as a source of data using automated web scraping implemented in Python in a Jupyter Notebook environment. After collection, the data were preprocessed systematically, including removal of duplicates. The resulting data constitute a structured dataset, which can be reused in machine learning, economic efforts, and policy-related studies. JUCars-2024 provides a publicly available, reproducible tool, which assists in price forecasting, market classification, modeling of the Jordanian used car market.

Specifications Table

**Table:** 

Subject	Computer Sciences
Specific subject area	*Table (CSV file)*
Type of data	*Raw, cleaned*
Data collection	*Automated data collection using custom Python web-scraping scripts that retrieved publicly available used vehicle advertisements from online car listing platforms*
Data source location	*Jordan*
Data accessibility	Repository name: Mendeley Data Data identification number: 10.17632/ddcz486x5t2 Direct URL to data: JUCars-2024: A Dataset of Used Car Listings in Jordan - Mendeley Data [1]
Related research article	*None*

## 1 Value of the Data

• The dataset provides a structured and reproducible profile of the Jordanian used car market.
• It supports the development of machine learning models that can predict the price and analyze demand.
• The dataset can be used by researchers to compare automotive markets across regions.
• Industry experts and policymakers can use the data to analyze price behavior and market trends.
• In addition, the dataset can support benchmark tasks such as supervised price prediction, feature importance analysis for vehicle attributes, and exploratory studies of market heterogeneity across cities and fuel types. These use cases illustrate how JUCars-2024 can serve as a reusable empirical foundation for data-driven automotive market research without imposing analytical assumptions. For instance, the dataset can support hedonic pricing models aimed at estimating the marginal contribution of vehicle characteristics (e.g., mileage, fuel type, transmission, regulatory status) to listing prices in the Jordanian market. Additionally, the inclusion of electric vehicle–specific attributes (battery capacity and range) enables exploratory analysis of EV market penetration and pricing dynamics. The city-level information further allows researchers to investigate geographic price dispersion and urban concentration patterns across different regions of Jordan.

## 2 Background

The Jordanian used car market plays an important role in providing affordable transportation due to high vehicle import taxes and increasing vehicle prices. Despite its importance, publicly available datasets capturing this market remain limited. This dataset has been assembled to fill this gap through systematic collection and organization of used car advertisements from online platforms. The dataset facilitates data-driven research by recording vehicle qualities and prices, and enhances transparency in the analysis of the automotive market.

## 3 Data Description

The dataset is provided as a structured CSV file of used car listings. The dataset contains 10,921 used car listings described by 22 features. The main attributes include brand, model, year, price, vehicle mileage, vehicle fuel type, transmission type and geographical location. Each record represents an individual listing collected during 2024. Table 1 provides a detailed description of the attributes included in the JUCars-2024 dataset.

**Table 1:** Description of attributes in the JUCars-2024 dataset.

Column name	Description
price_value_JOD	Listing price of the vehicle expressed in Jordanian Dinar (JOD)
make	Vehicle manufacturer or brand name
model	Specific model designation of the vehicle
year	Manufacturing year of the vehicle
mileage_km	Total distance driven by the vehicle, measured in kilometers
mileage_text	Mileage information as reported in the original online listing
body_type	Vehicle body classification (e.g., sedan, SUV, hatchback)
condition	Binary indicator specifying whether the vehicle is listed as new or used
transmission	Type of transmission system (manual or automatic)
fuel_type	Type of fuel used by the vehicle
engine_type	Engine or powertrain type (e.g., petrol, hybrid, electric)
color	Exterior color of the vehicle
interior_color	Interior color of the vehicle
seats	Number of seats available in the vehicle
city	City in which the vehicle is listed for sale
feat_insurance	Indicator of whether the vehicle has valid insurance coverage
feat_carcustoms	Indicator of whether customs duties have been settled
feat_license	Indicator of whether the vehicle is officially licensed
feat_paintcondition	Reported condition of the vehicle’s paintwork
feat_body_condition	Reported condition of the vehicle body
feat_battery_capacity	Battery capacity of electric vehicles, measured in kilowatt-hours (kWh)
feat_battery_range	Estimated driving range of electric vehicles, measured in kilometers

**Fig. 1:**
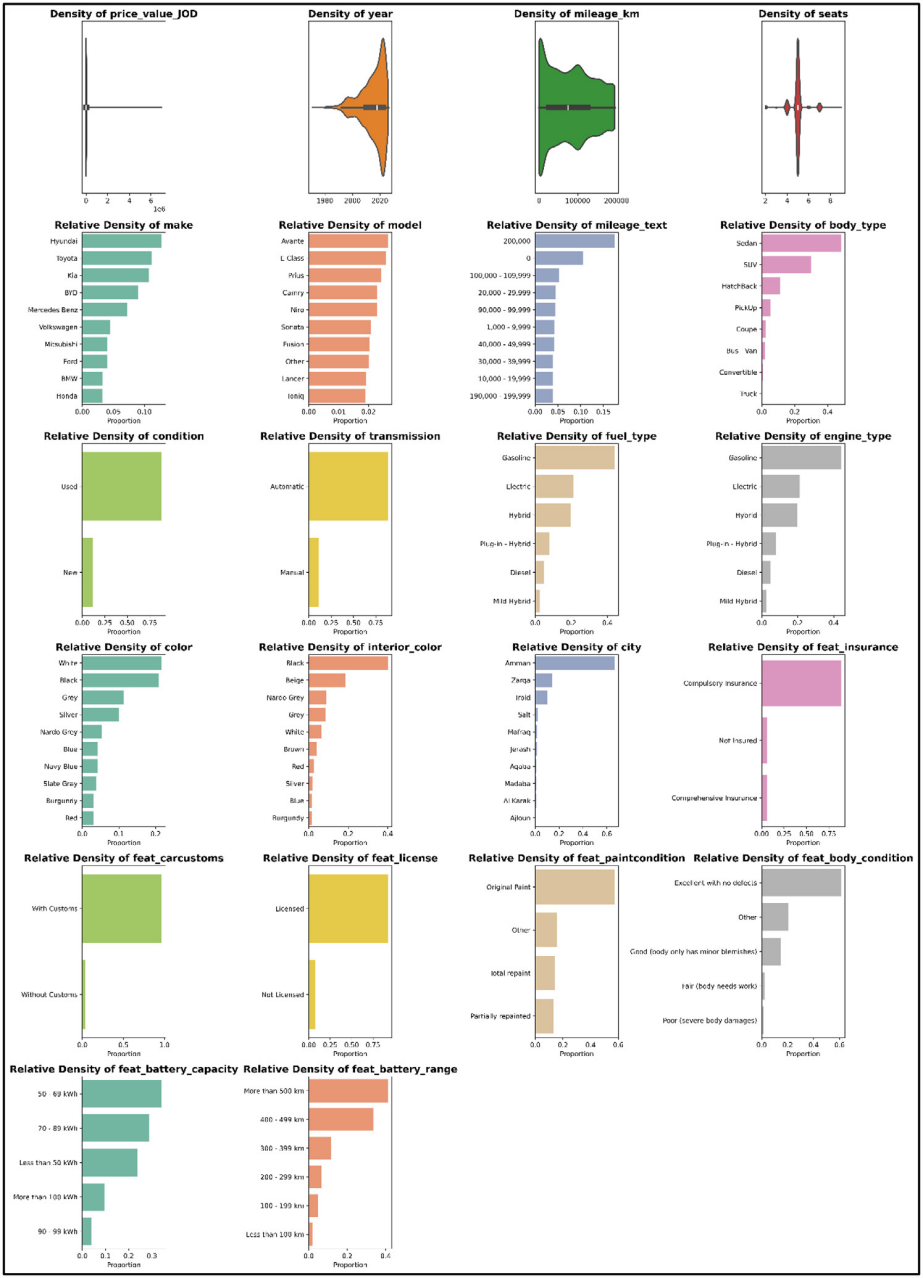
Density distributions of all attributes included in the JUCars-2024 dataset.

Fig. 1 shows density distributions of the attributes in the JUCars-2024 data through visualization based on the characteristics of the data. In case of numeric attributes, the violin plots that include box plots were used to demonstrate the entire distribution of values, such as central tendency, dispersion, and data density. In the case of categorical and binary attributes, normalized frequency bar plots have been employed to describe the relative density of most frequent categories. This dual visualization technique offers a descriptive summary of the dataset while ensuring that each attribute is represented using an appropriate statistical measure. The purpose of this figure is to promote transparency and reuse of the dataset, by allowing the readers to rapidly determine the variability and the composition of the gathered data.

Fig. 1 illustrates the statistical distributions of the price attribute, which appears as a non-symmetric distribution with an extended range toward higher values. The distributions of the remaining numeric attributes, such as manufacturing year and mileage, are also presented, showing their value ranges and dispersion within the dataset. For categorical and binary attributes, the visualizations display the relative frequency of categories based on their occurrence in the data, including fuel types, transmission modes, and regulatory status indicators. Overall, the figure provides a structured descriptive representation of the attribute distributions and the dataset composition as recorded, without introducing interpretive analysis or inferred conclusions.

Regarding data completeness, some attributes are not available for all listings, reflecting the realistic nature of online marketplace data. Battery-related attributes (feat_battery_capacity and feat_battery_range) are present only for electric vehicles and therefore exhibit high proportions of missing values (approximately 80 %). Mileage information (mileage_km) contains missing values in about 22 % of the records, primarily due to incomplete reporting in original listings. Several condition-related indicators, such as feat_paintcondition and feat_body_condition, show missingness levels of approximately 16–17 %. Price information (price_value_JOD) is missing in around 11 % of listings where sellers did not disclose a numeric price. These missing values were retained in the dataset to preserve transparency and reflect the original market reporting behavior.

Table 2 presents a descriptive summary of the main numeric variables in the Jordanian used car dataset, including price (price_value_JOD), manufacturing year (year), mileage (mileage_km), and number of seats (seats). The table reports the number of available values (count), mean, median, minimum (min), maximum (max), and the 25th and 75th percentiles. Specifically, the average car price is 15,758 JOD, with a median of 9900 JOD, ranging from 550 to 7000,000 JOD. The average manufacturing year is 2015, with a median of 2018, ranging from 1971 to 2026. The presence of a small number of listings reporting model years beyond the primary data collection period (e.g., 2026) may reflect the fact that data acquisition extended until the end of 2024, in addition to possible early model releases, pre-order listings, or seller-reported entries on online platforms. Mileage averages 78,727 km, with a median of 74,999 km, and ranges from 0 to 194,999 km. The number of seats has an average of 4.97, a median of 5, and ranges from 2 to 9. These descriptive statistics provide a clear overview of data distribution and variability, serving as a foundation for further analyses, such as hedonic pricing models or studies of electric vehicle adoption in Jordan.

**Table 2:** Descriptive Statistics of Numeric Variables in the Jordanian Used Car Dataset.

Attribute	Count	Mean	Median	Min	25 %	50 %	75 %	Max
price_value_JOD	9739	15,758.01	9900.0	550.0	4400.0	9900.0	16,900.0	7000,000.0
year	10,282	2015	2018	1971	2010	2018	2022	2026
mileage_km	8493	78,727.10	74,999.0	0.0	24,999.0	74,999.0	124,999.0	194,999.0
seats	10,277	4.97	5	2	5	5	5	9

## 4 Experimental Design, Materials and Methods

Information was gathered on various leading online Jordanian marketplaces that focus on advertisements of used cars, which are considered to be the leading sites of selling and purchasing vehicles in the Jordanian market. The included platforms were OpenSooq [2], Dubizzle [3], Motory [4], AutoBeeb [5], and Dooz [6], which include detailed listings of vehicle specifications, pricing and even geographical location of the advertisements. All these platforms are the most commonly used online sources of used car trading in Jordan and thus provide wide coverage of the used car market in the country.

Data acquisition was performed through repeated scraping sessions throughout 2024, approximately at intervals of one to two months. The timing of each session was guided by observable updates and listing activity on the selected platforms.

Automated web scraping was used to collect the data and was written in the Python language and ran in a Jupyter Notebook. The scraping process included browsing listing pages, scraping detailed vehicle attribute pages and iterating through the paginated listings, in order to cover all available advertisements on the chosen platforms.

At the preprocessing phase, the issue of duplicate advertisements was handled at two levels. To begin with, using the same platform, the identical advertisements were seen to reoccur at various times. Each platform-generated advertisement identifier was unique, and only the latest copy of any given advertisement was kept. Second, cross-platform duplicates were identified by matching completely identical records using core vehicle attributes such as manufacturer, model, manufacturing year, price, mileage, and location. Only one record was retained for exact cross-platform matches in the final dataset.

Also, the standardization of the price values occurred by converting all the reported prices into one single numeric form in terms of Jordanian Dinar (JOD), and the textual symbols and formatting discrepancies were eliminated. These steps were followed and the cleaned and structured data were saved in CSV format.

Beyond duplicate detection and basic format standardization, no statistical outlier filtering or implausible value removal was applied. The dataset intentionally preserves the original variability of marketplace listings in order to reflect real-world market behavior and allow researchers to apply their own validation or filtering procedures depending on the intended analytical use.

The scraping and preprocessing scripts are maintained by the authors. While the full code is not currently publicly archived, it can be made available upon reasonable academic request to support methodological transparency and reproducibility.

In addition, a concise data dictionary has been added to the Mendeley Data repository to enhance long-term usability. This supplementary file provides descriptions of all variables, data types, possible values, and relevant notes, facilitating understanding and reproducibility for future research.

## Limitations

The dataset is limited to listings collected within a single calendar year (2024) and may be subject to platform-related bias in terms of urban areas and the popularity of particular vehicle models.

It is important to emphasize that the dataset represents advertised listing prices rather than completed transaction prices. Therefore, the recorded prices reflect seller expectations and market offerings at the time of advertisement, and may differ from final negotiated sale prices. Researchers using the dataset for economic modeling or price inference should take this distinction into consideration.

## Ethics Statement

The authors confirm that this study does not involve human participants or animal experiments. All data were obtained from publicly accessible online vehicle advertisements published on car listing platforms operating in Jordan.

Data collection was conducted through automated web scraping of publicly available web pages in accordance with the Terms of Service (ToS) and publicly stated usage policies of the source websites at the time of data acquisition. No authentication, registration, login credentials, or circumvention of technical restrictions (e.g., paywalls or access controls) were involved during the scraping process. Responsible data collection practices, including rate limiting, were followed, and no platforms with explicit prohibitions against data scraping or data redistribution were included.

The dataset contains only vehicle-related, factual, and non-copyrightable information, such as prices, technical specifications, listing dates, and locations at the city level. It does not reproduce proprietary media content, website layouts, images, trademarks, or copyrighted textual descriptions.

With respect to privacy, no personal, sensitive, or identifiable information related to individual sellers or users (e.g., names, phone numbers, email addresses, account identifiers, or user profiles) was collected, stored, or shared. All potential personal identifiers were systematically excluded during preprocessing. Consequently, the dataset is fully anonymized, and no additional anonymization procedures were necessary.

The dataset is shared exclusively for research and academic reuse purposes and complies with the ethical standards and publication requirements of Data in Brief.

## CRediT Author Statement

**Nisrean Thalji:** Conceptualization, Methodology, Software, Investigation, Data Curation, Formal analysis, Writing – Original Draft, Visualization. **Khalid Tahat:** Conceptualization, Validation, Writing – Review & Editing, Supervision, Project administration.

## Data Availability

Mendeley DataJUCars-2024: A Dataset of Used Car Listings in Jordan (Original data).

## Appendix Supplementary materials

Image, application 1
